# Development of a peptide-siRNA nanocomplex targeting NF- κB for efficient cartilage delivery

**DOI:** 10.1038/s41598-018-37018-3

**Published:** 2019-01-24

**Authors:** Huimin Yan, Xin Duan, Hua Pan, Antonina Akk, Linda J. Sandell, Samuel A. Wickline, Muhammad Farooq Rai, Christine T. N. Pham

**Affiliations:** 1 Department of Medicine, Washington University School of Medicine, St. Louis, MO USA; 2 Department of Orthopaedic Surgery, Washington University School of Medicine, St. Louis, MO USA; 3 Department of Cardiovascular Sciences, University of South Florida Health Heart Institute, Morsani School of Medicine, Tampa, FL USA; 4 Department of Cell Biology & Physiology, Washington University School of Medicine, St. Louis, MO USA; 5 Department of Pathology and Immunology, Washington University School of Medicine, St. Louis, MO USA

## Abstract

Delivery of therapeutic small interfering RNAs (siRNAs) in an effective dose to articular cartilage is very challenging as the cartilage dense extracellular matrix renders the chondrocytes inaccessible, even to intra-articular injections. Herein, we used a self-assembling peptidic nanoparticle (NP) platform featuring a cell penetrating peptide complexed to NF-κB p65 siRNA. We show that it efficiently and deeply penetrated human cartilage to deliver its siRNA cargo up to a depth of at least 700 μm. To simulate osteoarthritis *in vitro*, human articular cartilage explants were placed in culture and treated with IL-1β, a cytokine with known cartilage catabolic and pro-inflammatory effects. Exposure of peptide-siRNA NP to cartilage explants markedly suppressed p65 activation, an effect that persisted up to 3 weeks after an initial 48 h exposure to NP and in the presence of continuous IL-1β stimulation. Suppression of IL-1β-induced p65 activity attenuated chondrocyte apoptosis and maintained cartilage homeostasis. These findings confirm our previous *in vivo* studies in a murine model of post-traumatic osteoarthritis and suggest that the ability of peptide-siRNA NP to specifically modulate NF-κB pathway, a central regulator of the inflammatory responses in chondrocytes, may potentially mitigate the progression of cartilage degeneration.

## Introduction

Osteoarthritis (OA) represents the most common form of arthritis and is a major cause of morbidity in the aging population. The annual health care burden for OA is $185 billion based on 2007 data, reflecting its very high prevalence in society and its negative quality of life impact^1^. Despite its prevalence, there are few treatment options—beside intermittent intra-articular (IA) corticosteroid or hyaluronic acid injections that are of questionable benefits^2,3^ — and no disease-modifying OA drugs (DMOADs).

IA delivery of therapeutics is ideal for the treatment of OA, a disease that usually affects a few large joints. It offers direct access to the joint space, thus increasing local concentration of the drugs while limiting systemic side effects. Despite clear advantages and critical medical need, successful DMOAD development for IA delivery remains a challenge and none has successfully emerged in clinic. The reasons for this failure are multifold. Most drugs delivered IA are rapidly cleared from the joint cavity and have difficulty reaching chondrocytes residing in the avascular cartilage due to the dense extracellular matrix. In addition, primary OA is not a single disease but a syndrome with multiple etiologies that are still poorly understood.

Post-traumatic OA (PTOA) is a form of OA that develops after a joint injury and in which the nature and time of trauma is generally known. Approximately 12% of the overall OA burden can be traced to joint trauma^4^ but true prevalence for PTOA may be higher, given the long delay (10–15 years) between injury and end-stage PTOA^5^. Studies have documented a robust inflammatory response in the immediate aftermath of joint injury (i.e. during the first week and up to 2–3 months) that persists for months to years, albeit at a lower level^6^. This inflammatory response likely contributes to chondrocyte death, impaired cartilage repair and subsequent cartilage degeneration that constitute PTOA^6^.

We have developed a peptidic nanoparticle (NP) that suppresses NF-κB activities via small interfering RNAs (siRNAs). Using a murine model of non-invasive controlled injurious compressions of the knee joint that allowed the examination of cartilage responses at specific time points after trauma, we showed that joint injury led to NF-κB activation within hours. IA delivery, immediately after injury, of a NP comprising a peptide, p5RHH complexed to p65 siRNA suppressed NF-κB expression and significantly reduced two important early events: chondrocyte apoptosis and reactive synovitis^7^.

In anticipation of translating this technology to the point of care, we tested the efficacy of p5RHH-NF-κB p65 siRNA NP in human articular cartilage explants as a proof-of-concept that our platform can eventually translate to the clinic. To this end, we showed that the peptide-siRNA NP freely and deeply penetrated all layers of the much thicker human cartilage explants to deliver its siRNA cargo to the chondrocytes. In addition, the siRNA signal was detected in chondrocytes for up to 21 days^7^ and p65 suppression persisted for up to 3 weeks. These promising findings warrant further investigation into the long-term outcomes studies in rodent models of PTOA and further validation in a large animal model.

## Results

### NF-κB siRNA NP efficiently transfects human umbilical endothelial cells (HUVECs) and chondrocytes *in vitro*

The self-assembling p5RHH-siRNA NP was formulated as previously described^8^. Briefly, p5RHH (20 mM stock solution) was dissolved 1:400 in Opti-mem followed by the addition of a smart pool of 4 human p65 siRNAs (50 nM final concentration). The preparation was incubated at 37 °C for 40 min yielding peptide-siRNA NP that measured ∼55 ± 18 nm by wet mode atomic force microscopy^8,9^ and had a zeta potential of ~12 ± 0.7 mV^9^. In addition, we have already shown that this 40 min incubation exhibits maximal transfection efficiency *in vitro*^8^.

**Figure 1:**
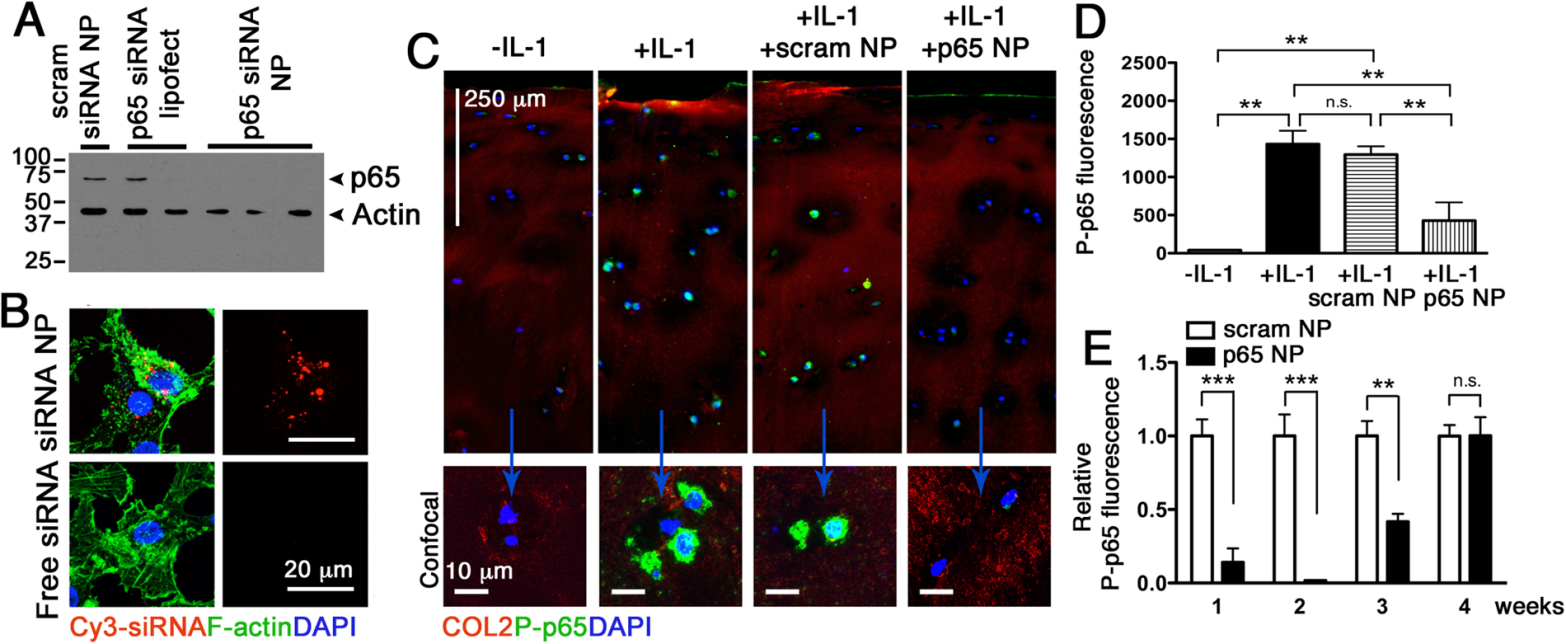
p5RHH-p65 siRNA NP transfects HUVECs, human chondrocytes, and cartilage explants to suppress p65. (**A**) HUVECs were cultured to 50% confluence and transfected with p65 siRNA (RELA SMART pool, 100 μM F.C.) or scrambled siRNA delivered byLipofectamine or peptide-siRNA nanocomplex. p65 expression was detected by Western blot. Actin served as protein loading control. (**B**) Human primary chondrocytes were cultured as detailed in Materials and Methods and transfected with p5RHH-Cy3–labeled siRNA NP or free Cy3-labeled siRNA for 48 h. Cy3-siRNA (red) in chondrocytes was visualized by confocal microscopy. IL-1β stimulated human cartilage explants were incubated with p5RHH-p65 siRNA (p65 NP with 500 nM siRNA) or scrambled siRNA (scram NP) for 48 h after which the excess NP was washed off and cartilage stained and analyzed or cultured in the presence of IL-1β (10 ng/ml) for a total of 4 weeks. (**C**) Immunofluorescence of p65 (green) in cartilage sections at day 7 following different treatments. Bottom row: representative 2D confocal images at higher magnification. (**D**) Mean fluorescence of P-p65 per chondrocyte (from z-stack confocal images) 48 h after IL-1 β and NP exposure. (**E**) Relative mean fluorescence of P-p65 per chondrocyte at different time points. Mean P-p65 fluorescence in cartilage exposed to scrambled siRNA NP was set at 1. Mean reflects analysis of 5–8 areas per explant, each including 3–6 cells; 3–5 individual cartilage explants were analyzed per treatment per time point. **P < 0.01, ***P < 0.001; n.s. not significant. COL2 (red), type II collagen; DAPI (blue) stained nuclei.

We first tested the ability of p5RHH-p65 siRNA NP to knockdown p65 expression in HUVECs. Delivery of human p65 siRNA NP led to marked decrease in p65 expression in these cells and exhibited similar or superior transfection efficiency compared with Lipofectamine (Fig. 1A). NP containing the scrambled siRNA sequences on the other hand did not silence p65 expression in HUVECs (Fig. 1A). Next, we examined the uptake of p5RHH-Cy3-labeled siRNA NP in human primary chondrocytes. We observed that p5RHH-siRNA NP, but not free Cy3-labeled siRNA, was taken up by the chondrocytes (Fig. 1B). These results confirmed the specificity of the p65 siRNAs as well as the transfection efficiency of the NP.

### NF-κB siRNA NP suppresses p65 in IL-1β-treated human cartilage explants

To enhance the expression of NF-κB, we exposed human articular cartilage explants to IL-1β, a cytokine with known catabolic and a pro-inflammatory effects on cartilage^10^. We confirmed that IL-β exposure led to significant activation of p65, as evidenced by an increase in its phosphorylation and nuclear translocation (Fig. 1C,D). Treatment with NF-κB NP markedly suppressed IL-1β-mediated p65 activation, an effect that was apparent 48 h after NP exposure (Fig. 1C) and lasted up to 3 weeks after the initial exposure (Fig. 1E).

### NF-κB siRNA NP preserves chondrocyte viability and maintains cartilage homeostasis

**Figure 2:**
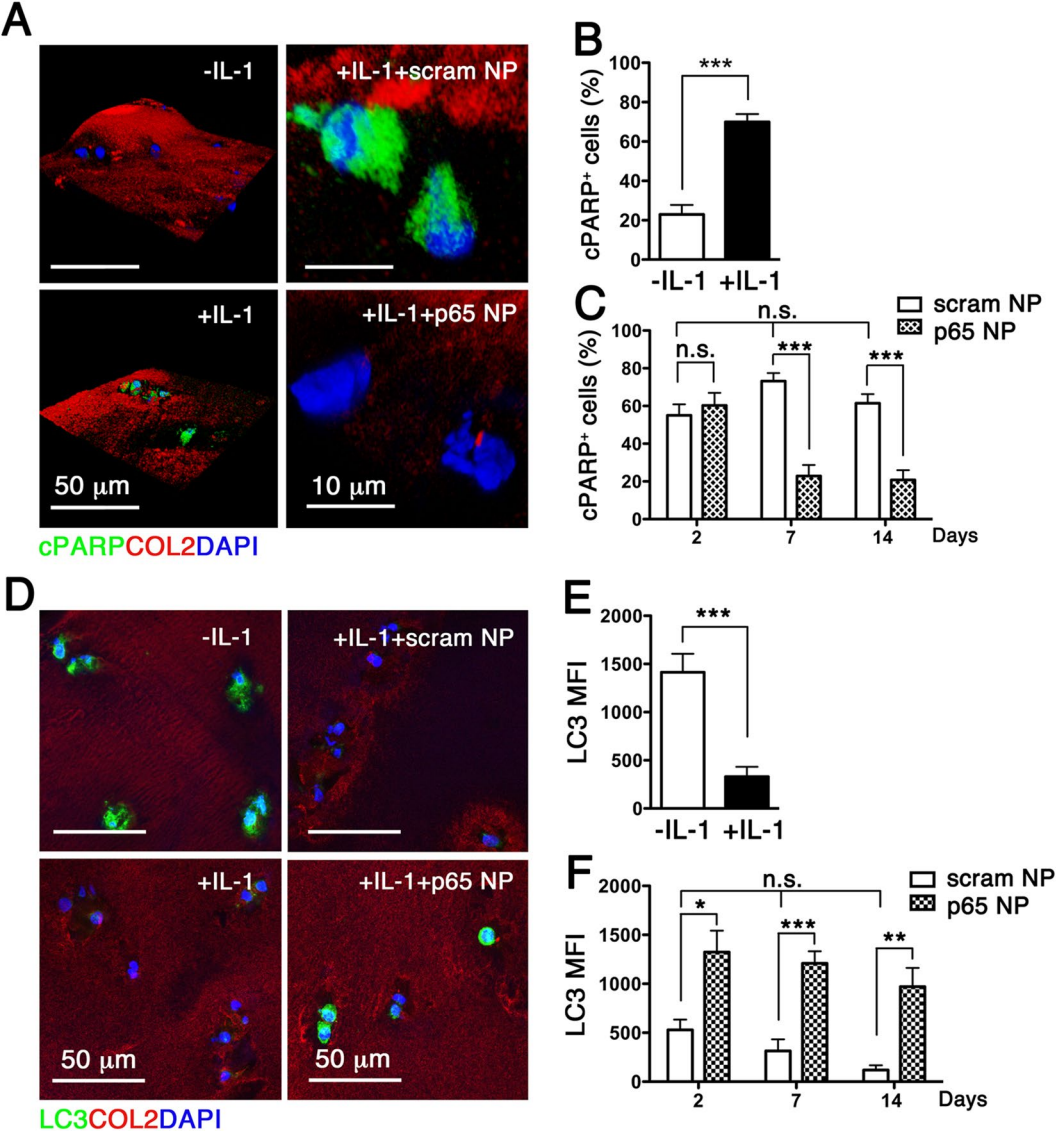
p5RHH-p65 siRNA NP attenuates chondrocyte apoptosis and maintains cartilage homeostasis. IL-1β-treated human cartilage explants were incubated with p5RHH-p65 siRNA NP (p65 NP) or scrambled siRNA NP (scram NP) as detailed above. (**A**) Immunofluorescence of cleaved PARP (cPARP) (green) in cartilage sections at day 7. (**B**) Number of cells with nuclear cPARP at 48 h and (**C**) at 14 days after IL-1 β exposure and NP treatment. **(D)** Immunofluorescence of LC3 in cartilage sections without and with IL-1β exposure at day 7. (**E**) Mean fluorescence of LC3 per chondrocyte after exposure to IL-1β at day 7 and (**F**) at different time points after exposure to NP treatment. Mean reflects analysis of 5–8 areas, each including 3–6 cells, n = 3–5 individual cartilage explants were analyzed per treatment per time point. *P < 0.05, **P < 0.01, ***P < 0.001; n.s. not significant. COL2 (red), type II collagen; DAPI (blue) stained nuclei.

We have previously demonstrated that mechanical joint injury *in vivo* activates NF-κB signaling, leading to chondrocyte apoptosis^7^, a pathological feature of PTOA. NF-κB activation promotes the release of pro-inflammatory cytokines such as IL-1β, which has been shown to induce apoptosis in human articular chondrocytes^11^, thus providing a useful *in vitro* model of OA. Here we show that exposure of human cartilage explants to IL-1β indeed led to chondrocyte apoptosis, as evidenced by nuclear localization of poly(ADP-ribose) polymerase (PARP) C-terminal cleavage fragment (Fig. 2A,B). To determine whether NF-κB siRNA NP suppressed IL-1β-induced apoptosis of chondrocytes, we exposed human cartilage explants to p5RHH-p65 siRNA NP (500 nM) for 48 h while maintaining the explants under continuous IL-1β stimulation. Consistent with *in vivo* studies^7^, exposure of cartilage explants to NP did not affect cell viability at the articular surface (Fig. S1). In addition, p5RHH-p65 siRNA NP significantly inhibited nuclear localization of cleaved PARP (an indication of apoptosis), an effect that was evident one week after the NP exposure and lasted at least until week 2 (Fig. 2C).

**Figure 3:**
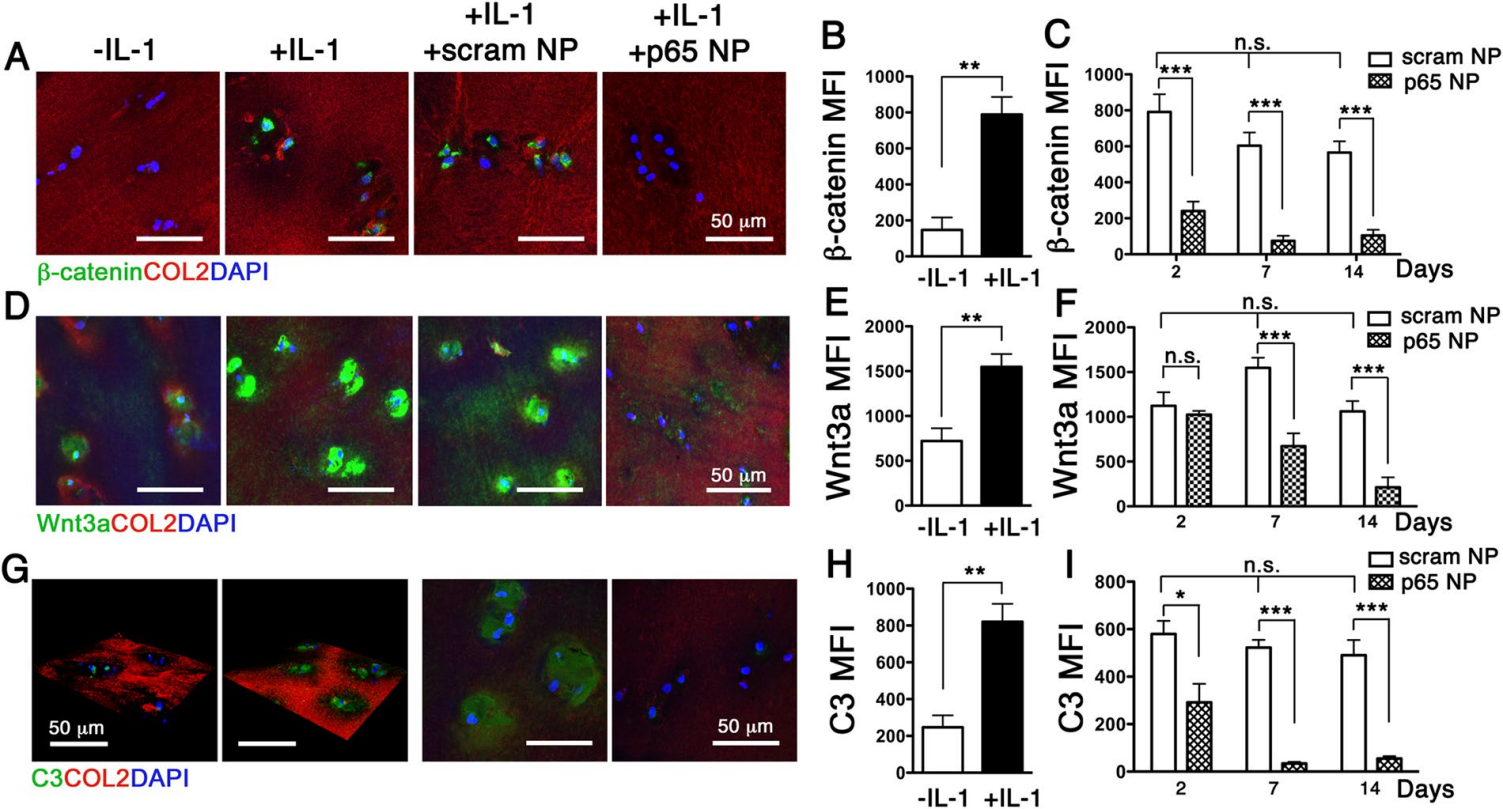
p5RHH-p65 siRNA NP modulates crosstalk between NF-κB and Wnt signaling/complement system. IL-1β stimulated human OA cartilage explants were incubated with p5RHH-p65 siRNA NP (p65 NP) or scrambled siRNA NP (scram NP) in the presence of IL-1β as detailed above for a total of 2 weeks. Immunofluorescence of β-catenin (**A**), Wnt3a (**D**), and C3 (**G**) in cartilage sections at day 7 without and with IL-1β treatment. Mean fluorescent intensity of β-catenin (**B**), Wnt3a (**E**), and C3 (**H**) per chondrocyte (from z-stack confocal images) 48 h following IL-1 β exposure. Mean fluorescent intensity (MFI) of β-catenin (**C**), Wnt3a (**F**), and C3 (**I**) per chondrocyte at different time points after exposure to NP treatment. Mean reflects analysis of 5–8 areas per explant, each including 3–6 cells; 3–5 individual cartilage explants were analyzed per treatment per time point. *P < 0.05, **P < 0.01, ***P < 0.001; n.s. not significant. COL2 (red), type II collagen; DAPI (blue) stained nuclei.

Normal chondrocytes express high level of the autophagy-related gene (*Atg*) called microtubule-associated protein light chain 3 (LC3) and its expression decreases with aging and OA^12^. Our data revealed that exposure of human cartilage explants to IL-1β significantly reduced the expression of LC3 (Fig. 2D,E). Treatment of the explants with p5RHH-p65 siRNA NP, but not the p5RHH -scrambled siRNA NP, preserved LC3 expression (Fig. 2F). Moreover, p5RHH-p65 siRNA NP blocked the nuclear translocation of β-catenin (Fig. 3A–C), the effector of canonical Wnt signaling, a pathway implicated in OA^13^. Consistent with these results we found that the expression of Wnt3a, the prototypical activator of the β-catenin-dependent canonical pathway^14^ was similarly suppressed (Fig. 3D–F).

### NF-κB modulates the expression of complement in cartilage

The complement system plays a central role in innate immunity and participates in many disease processes. Accumulating evidence suggests that complement activation contributes to OA pathogenesis^15^. Expression of many complement proteins, including C3, C4, and factor B is reported in OA cartilage, synovial membrane, and cultured chondrocytes^15^. Moreover their expression is amplified by IL-1β stimulation^15^. In contrast mice lacking in certain complement proteins, such as C5, are protected against OA development^16^. Consistent with published results we observed a marked upregulation of C3 in human cartilage explants with IL-1β stimulation (Fig. 3G,H). Treatment of cartilage explants with p5RHH-p65 siRNA NP significantly suppressed C3 expression (Fig. 3I). This finding uncovers a crosstalk between NF-κB signaling and complement regulation in a joint-associated tissue.

## Discussion

In previous studies, we have shown that the p5RHH-siRNA nanocomplex efficiently penetrated the dense extracellular matrix to deliver its siRNA cargo to all the layers of human articular cartilage^7^. We have also shown that fluorescently labeled siRNA persisted within chondrocyte lacunae for up to 3 weeks after the initial exposure to NP^7^. In this study, we show that an initial exposure of p5RHH-siRNA NP led to significant suppression of p65 expression in IL-1β-treated human cartilage explants, an effect that persisted for at least 3 weeks. Suppression of p65 expression was accompanied by maintenance of chondrocyte viability (evidenced by decrease in nuclear localization of cleaved PARP) and cartilage homeostasis (evidenced by preservation of LC3 expression). Serendipitously we also found that p5RHH-siRNA NP modulated the crosstalk between NF-κB signaling pathway and the complement system. Thus exploring the role of complement in the joint may, in the future, offer novel therapeutic strategies for OA treatment.

Much interest has recently focused on the role of inflammation and inflammatory cytokines in the pathogenesis of PTOA (and possibly primary OA). It is posited that an imbalance between inflammatory/catabolic and anabolic cytokines lead to cartilage degeneration, a hallmark of PTOA. Thus, blockade of catabolic cytokines such as TNF-α and IL-1β has gained attention as potential therapy. Indeed, IA administration of IL-1 receptor antagonist (IL1-ra) exhibits disease-modifying effects in a rodent model of PTOA^17^. In clinical studies, however, the administration of commercially available IL-1ra (Anakinra) did not show any therapeutic effects in established knee OA in humans^18^. These studies suggest that targeting IL-1β pathway is unlikely to impact the progression of OA once established. However, anti-NF-κB nanotherapy administered in the acute phase, post-joint injury may improve long-term primary outcomes of PTOA.

The NF-κB pathway is the central regulator of the inflammatory responses in synovium and chondrocytes^19^, leading to the expression of inflammation-related genes, including TNF-α, IL-1β, IL-6, nitric oxide (NO), matrix-degrading metalloproteases (MMPs), and the ADAMTS family of aggrecanases. NF-κB pathway thus represents an attractive therapeutic target for PTOA. However, the indiscriminate systemic blockade of NF-κB poses significant risks, given its central role in host immune responses. As targeted strategies to silence NF-κB only in affected joints via IA delivery might help avoid unwanted systemic effects, they are highly desirable. We have shown *in vitro* (in human cartilage explants) and *in vivo* (in rodent model of PTOA) that our p5RHH-siRNA nanoplatform targeting NF-κB can overcome the difficulties in therapeutic delivery to articular cartilage and promises real translational potential as a disease-modifying IA therapy in the treatment of PTOA. Long-term outcomes studies in rodent models of PTOA and further validation in a large animal model are currently underway in our laboratory. We acknowledge that targeting NF-κB alone may fail to modify the long-term outcomes of PTOA. If this were the case, we will explore targeting multiple pathways (3 or more) simultaneously, as our p5RHH peptide allows multiplexing option for multiple siRNAs.

## Methods

### p5RHH-siRNA NP Preparation

p5RHH peptide (VLTTGLPALISWIRRRHRRHC) provided by Genscript was dissolved at 20 mM in DNase-, RNase-, and protease- free sterile purified water (Cellgro) and stored in 10 μL aliquots at −80 °C before use. Human RELA SMART pool SiRNAs (a mixture of 4 different siRNAs at a ratio of 1:1:1:1; Cat# M-003533-02, Dharmacon) and ON-TARGETplus control Non-targeting pool siRNAs (Cat# D-001810-05, Dharmacon) were dissolved at 20 uM in DNase, RNase-, and protease- free sterile purified water (Cellgro) and stored in 10 μL aliquots at −80 °C until use.

For cell culture p5RHH siRNA NP was prepared by diluting p5RHH peptide (20 mM) 1:400 in Opti-MEM (Thermo Fisher Scientific), vortexed for 30 s followed by the addition of appropriate volume of RELA SMART pool or ON-TARGETplus control Non-targeting pool stock to achieve a peptide to siRNA ratio of 100 to 1. The mixture was incubated at 37 °C for 40 min with shaking.

For cartilage explant culture, the p5RHH siRNA NP was freshly prepared by mixing p5RHH peptide stock (20 mM) and siRNA (20 uM) to achieve a peptide to siRNA ratio of 1 to 10 in HBSS with Ca^2+^ and Mg^2+^ (Gibco, Life Technologies). The mixture was incubated on ice for 10 min and added directly to cartilage explants.

### HUVEC culture

HUVECs procured from Sigma-Aldrich were seeded at low density in 12-well plates and cultured in Endothelial Cell Growth Medium (Cat# 50306188, Thermo Fisher Scientific) to 50% confluence. On the day of experiment, HUVECs were washed with warm Opti-MEM and siRNA-p5RHH NP was added to cells and incubated for 6 h in 1 ml Opti-MEM per well. Unbound complexes were removed and HUVECs were cultured for 72 h under regular conditions. Lipofectamine (RNAiMAX, Cat# 13778-030, Invitrogen) was used according manufacturer instructions. Cells were lysed directly in SDS sample buffer transferred to tubes, boiled for 10 min and stored at −20 °C until use.

### Western blot analysis

Cell lysates were fractionated by SDS-PAGE under reducing condition and blotted with rabbit anti-human p65 (1:1000, Cat# 33034, Cell Signaling) for 2 h at RT followed by incubation with HRP-conjugated Donkey anti-rabbit (Cat# 711-035-152, Jackson ImmunoResearch Laboratories). The bands were visualized using a SuperSignal West Pico Kit (Cat# 34080, Thermo Scientific,). The membrane was stripped and re-probed with anti-actin antibody (1:500, Cat# A2103, Sigma-Aldrich) to control for protein loading.

### Human chondrocyte culture

All research involving human tissue was performed in accordance with relevant guidelines and regulations established by the Washington University in St. Louis Institutional Review Board (IRB) Committee. Human primary chondrocytes were isolated from the human cartilage obtained from discarded articular tissues harvested at the time of total knee arthroplasty from consented patients through a protocol approved by the IRB and all study participants provided written informed consent. The cartilage explants were maintained in 75 cm^2^ culture flasks in DMEM/F12 with 10% FBS, 100 U/ml penicillin, and 100 μg/ml streptomycin supplemented with 250 mg/L collagenase P and 250 mg/L Pronase for 15 h at 37 °C. The isolated cells were grown in 5% CO_2_ and 90% humidity at 37 °C. Cell viability was evaluated routinely by trypan blue exclusion method using a hemocytometer. Primary human chondrocytes were seeded on chamber slides and transfected with p5RHH-Cy3–labeled siRNA NP for 48 h, after which the chambers were washed with PBS, the cells were fixed and stained with F-actin (1:1000, Cat# ab176753, Abcam). Chamber slides were mounted with VECTASHIELD mounting medium with DAPI (Cat# H-1200, Vector Laboratories) and images were acquired using confocal microscopy.

### Human cartilage explant culture

Human cartilage explants were obtained as described above. The de-identified cartilage tissues were washed several times with HBSS containing antibiotics then incubated in DMEM/F12 (1:1) medium containing 10% FBS, penicillin/streptomycin (100 U/0.1 mg/mL), amphotericin B (0.25 μg/mL), and ciproflaxin (10 μg/mL) in a 6-well plate at 37 °C and 5% CO_2_ for 2–3 days. The explants were then transferred to a 96-well plate and were treated with IL-1β (10 ng/ml, Cat# PHC0815, Thermo Fisher Scientific Inc.) and subsequently exposed to p5RHH-p65 siRNA or scrambled siRNA (at a final concentration of 500 nM of siRNA) for 48 h. The excess NP was washed off after 48 h incubation and the explants were cultured in medium supplemented with IL-1β (10 ng/ml) for up to 28 days. The cartilage explants were then embedded in Tissue-Tek Optimal Cutting Temperature (O.C.T.) compound (Sakura Finetek USA, Inc.) and sectioned for analysis.

### Immunofluorescence and confocal microscopy

Frozen sections (9 μm thick) were fixed and permeabilized with 0.5% Tween-20/PBS if needed and blocked with 8% BSA or Tyramide Signal Amp (TSA) blocking solution for 1 h at room temperature. After blocking the sections were incubated with the primary antibodies: anti-LC3 (1:100, Cat# L7543, Sigma-Aldrich), phospho-p65 (1:100, ab28856, Abcam), β-catenin (1:100, Cat# ab16051, Abcam), cleaved PARP (1:100, Cat# 9544, Cell Signaling), WNT16 (1:100, Cat# LS-A9629, LifeSpan Biosciences), WNT3A (1:100, Cat# OABF00803, Aviva Systems Biology), C3 (1:200, Cat# 55463, MP Biomedicals) overnight at 4 °C. After washing the sections were incubated with the corresponding HRP-conjugated secondary antibodies. All the sections were applied with Biotinyl Tyramide working solution (1:100) for 7 min to enhance the specific signal and subsequently incubated with Alexa Fluor 488-conjugated–streptavidin (1:200, Cat# S-11223, Molecular Probes) for 1 h. The sections were probed with COL2 antibody (1:200, generously provided by L. J. Sandell and M. F. Rai, Washington University, St. Louis) followed by TRITC-conjugated anti-rat secondary antibody (1:100, Cat# 712-295-153, Jackson ImmunoResearch) and counterstained with DAPI (1:1,000, Vector Laboratories). The images were acquired with ZEISS LSM 880 Confocal Laser Scanning Microscope and 15 to 20 cells per section and 3–4 sections were analyzed with software ZEN. The data was presented as the mean fluorescent intensity per cell or ratio of positive cells.

### Statistics

Comparisons between multiple groups (≥3) were performed by one-way ANOVA followed by Bonferroni’s correction for multiple comparisons was performed. Differences between experimental groups at a P value of <0.05 and a statistical power of 0.80 were considered significant.

## Supplementary information

Supplementary Information

## Data Availability

Data generated in this current study are available from the corresponding author on reasonable request. The materials used to formulate nanoparticles are obtained from commercial vendors and methods are detailed in this study and through public searchable databases.
