# Supplementary material for: Development of a peptide-siRNA nanocomplex targeting NF- κB for efficient cartilage delivery
Source: Sci Rep. 2019 Jan 24;9:442. doi: 10.1038/s41598-018-37018-3 (PMC6345850; doi:10.1038/s41598-018-37018-3)
Supplement: Supplementary file 1 — Supplementary Information [file 41598_2018_37018_MOESM1_ESM.pdf]

Development of a peptide-siRNA nanocomplex targeting NF-κB for efficient cartilage delivery

Huimin Yan[1], Xin Duan[2], Hua Pan[3], Antonina Akk[1], Linda J. Sandell[2], Samuel A. Wickline[3], Muhammad Farooq Rai[2,4], and Christine T.N. Pham[1,5,*]

[1]Department of Medicine, Washington University School of Medicine, St. Louis, MO

[2]Department of Orthopaedic Surgery, Washington University School of Medicine, St. Louis, MO

[3]Department of Cardiovascular Sciences, University of South Florida Health Heart Institute, Morsani School of Medicine, Tampa, FL

[4]Department of Cell Biology & Physiology, Washington University School of Medicine, St. Louis, MO

[5]Department of Pathology and Immunology, Washington University School of Medicine, St. Louis, MO

*Address correspondence to: Christine Pham, Washington University School of Medicine, 660 South Euclid Avenue, Box 8045, Saint Louis, MO 63110, USA. Phone: 314.362.9043; Fax: 314.454.1091; Email: cpham@wustl.edu

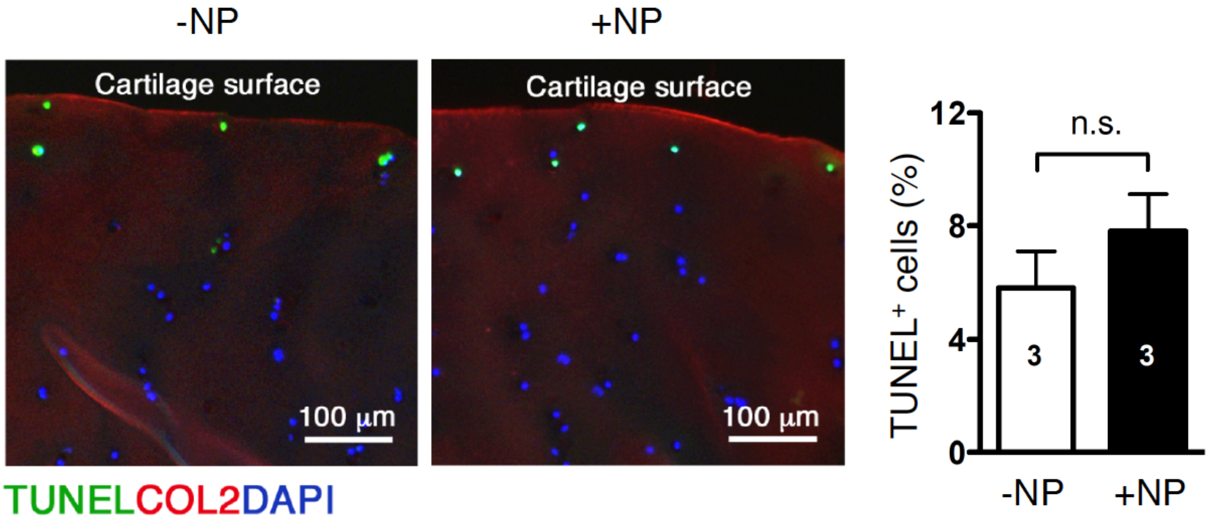

Figure S1. P5RHH-p65 siRNA NP does not negatively affect chondrocyte viability at the cartilage surface. Human cartilage explants were incubated in culture medium without or with p5RHH-p65 siRNA NP for 48 h. Excess NP was washed off and cartilage sections were examined for cell viability using TUNEL staining (green). Exposure to NP did not significantly affect cell viability. COL2 (red), type II collagen; DAPI (blue) stained nuclei. N = 3 explants per treatment type.

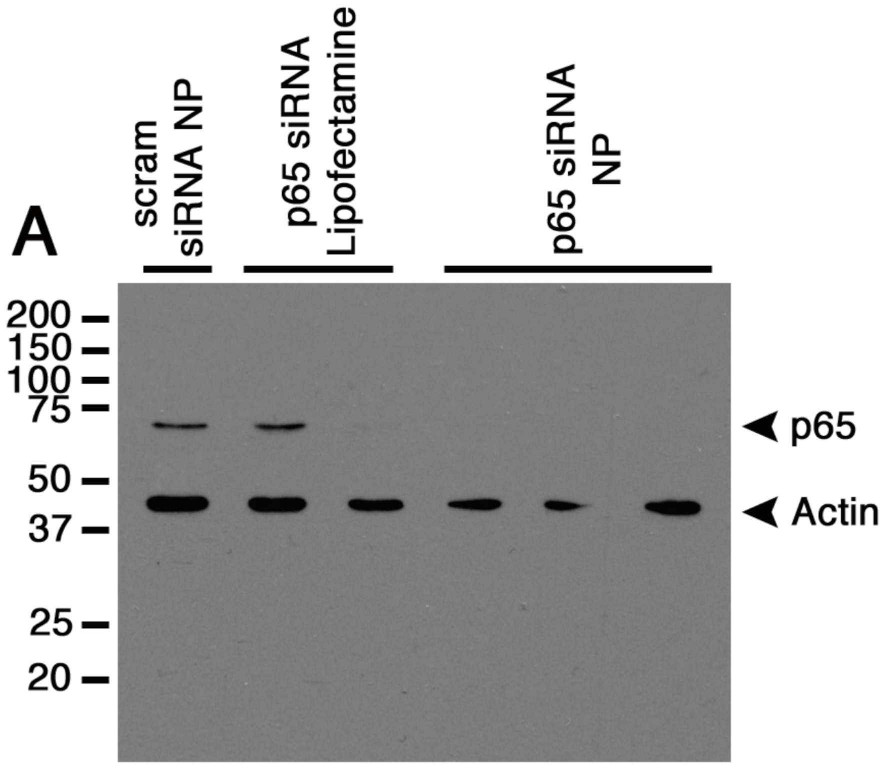

Figure 1A. Uncut gel
